# Difficulties faced by physicians from four European countries in rebutting antivaccination arguments: a cross-sectional study

**DOI:** 10.1136/bmjph-2023-000195

**Published:** 2024-03-12

**Authors:** Dawn Holford, Philipp Schmid, Angelo Fasce, Amanda Garrison, Linda Karlsson, Frederike Taubert, Pierre Verger, Stephan Lewandowsky, Harriet Fisher, Cornelia Betsch, Fernanda Rodrigues, Anna Soveri

**Affiliations:** 1 School of Psychological Science, University of Bristol, Bristol, UK; 2 Centre for Language Studies, Radboud Universiteit, Nijmegen, The Netherlands; 3 Institute for Planetary Health Behaviour, University of Erfurt, Erfurt, Germany; 4 Health Communication, Department of Implementation Research, Bernhard-Nocht-Institute for Tropical Medicine, Hamburg, Germany; 5 Faculty of Medicine, Universidade de Coimbra, Coimbra, Portugal; 6 Faculté des Sciences Médicales et Paramédicales, Observatoire Regional de la Sante Provence-Alpes-Cote d'Azur, Marseille, France; 7 Department of Clinical Medicine, University of Turku, Turku, Finland; 8 Department of Psychology, University of Potsdam, Potsdam, Germany; 9 School of Psychological Science, The University of Western Australia, Perth, Western Australia, Australia; 10 National Institute for Health Research Health Protection Research Unit (NIHR HPRU) in Behavioural Science and Evaluation (BSE), University of Bristol, Bristol Medical School, Bristol, UK

**Keywords:** Public Health, Community Health, Communicable Disease Control

## Abstract

**Introduction:**

Physicians play a critical role in encouraging their patients to get vaccinated, in part by responding to patients’ concerns about vaccines. It is, therefore, important to understand what difficulties physicians have in dealing with different concerns they may encounter. The aim of this article was to determine physicians’ perceptions of difficulties in rebutting different antivaccination arguments from patients using data collected as part of a cross-sectional, cross-national questionnaire on physicians’ vaccine attitudes and behaviours.

**Methods:**

Physicians in 4 European countries (Finland, Germany, France and Portugal, total n=2718) rated 33 different arguments, chosen to represent 11 different psychological motivations underlying vaccine hesitancy, in terms of their perceptions of how difficult each argument would be to rebut.

**Results:**

Across all countries, physicians perceived arguments based on religious concerns and ‘reactance’ (ie, resistance to perceived curbs of freedom) to be the most difficult to rebut, whereas arguments based on patients’ distorted perception of the risks of disease and vaccines were perceived to be the easiest. There were also between-country differences in the level of perceived difficulty of argument rebuttal. Physicians’ perceived difficulty with rebutting arguments was significantly negatively correlated with their vaccine recommendation behaviours and their preparedness for vaccination discussions.

**Conclusions:**

Physicians may feel better equipped to counter arguments that can be rebutted with facts and evidence but may struggle to respond when arguments are motivated by psychological dispositions or values.

**WHAT IS ALREADY KNOWN ON THIS TOPIC**

- Physicians play important roles in rebutting flawed antivaccination rhetoric and misinformation that patients have encountered.

**WHAT THIS STUDY ADDS**

- In this study, 2718 physicians from 4 European countries found it most difficult to rebut antivaccination arguments stemming from religious concerns and a resistance towards curbs on freedom, while it was least difficult to rebut arguments stemming from distorted risk perceptions. Physicians who perceived higher difficulty with argument rebuttal reported lower vaccine recommendation behaviour and lower proactivity and preparedness for vaccine-related conversations.

**HOW THIS STUDY MIGHT AFFECT RESEARCH, PRACTICE OR POLICY**

- Understanding which types of antivaccination arguments are difficult for physicians to rebut can inform the development of targeted vaccine communication training for healthcare professionals.

##  Introduction

Physicians play a critical role in encouraging vaccine uptake.^1^ They are trusted providers of healthcare services^2 3^ with the opportunity to raise the topic of vaccination and, therefore, are in a good position to discuss vaccines with patients and their caregivers.^2 4^ These discussions will often go beyond simply recommending a vaccine.^5^ While a physician’s recommendation to vaccinate can be influential in itself,^6 7^ recommending vaccines to patients who are hesitant requires an understanding of patients’ objections to be able to address them effectively.^1^ In dealing with patients’ objections, physicians may need to rebut vaccine misinformation and other misconceptions.^8^,^11^ However, it can be difficult for a physician to confidently approach such a conversation, for example, because patients may not be receptive to facts or corrections.^12 13^ This may be specifically relevant as physicians and the World Health Organization (WHO) have expressed concerns regarding the consumption of misinformation from easily available online sources^14^ and a shift in doctor–patient interactions where patients became more willing to negotiate and argue with the former unchallenged traditional health authority.^15^ These factors could affect the physician’s propensity to continue the conversation or even recommend vaccines to the patient.^16^ Therefore, it is important to understand the difficulties physicians have with different patient objections and how these relate to their preparedness to address objections and recommend vaccines.

In this paper, we present data from a large survey of physicians across four European countries (France, Finland, Portugal and Germany) that show the variation in difficulties physicians face in rebutting arguments against vaccines that can be rooted in different psychological motivations (ie, ‘attitude roots’^17 18^), and how physicians’ difficulty at refuting arguments is associated with their vaccine recommendation behaviours and their preparedness for discussing vaccines.

### Difficulties in dealing with vaccine resistance

Discussions about vaccines often do not occur in isolation—they are part of a wider healthcare system that builds on the ongoing relationship between physicians and their patients. This relationship can be helpful for encouraging vaccine recommendations, as it positions physicians as a trusted source of information.^3 19^ By the same token, physicians may find it more difficult to discuss vaccines if they worry that it will affect a longer-term relationship with their patients.^8 20^ Previous surveys of physicians responsible for delivery of the human papillomavirus (HPV) vaccination programmes in Australia and the USA found that physicians felt conversations with strongly hesitant patients (or their caregivers) to be especially challenging^8^ and many healthcare professionals (HCPs) felt they could not change their patients’/caregivers’ minds.^16^ Difficulties with vaccine conversations can influence vaccine recommendation behaviours.^3 16^ For example, healthcare providers who felt less confident to effectively address their patients’ concerns were less likely to routinely recommend the HPV vaccine to vaccine-eligible young people.^16^

While it is impossible to know in advance what concerns a specific patient may raise, research has shown that arguments opposing vaccines tend to cluster around a finite set of themes.^21^,^23^ ‘Arguments’ in this context refer to the propositions put forth by patients as a rationale for not having a vaccine. Many different studies have documented these arguments, resulting in a rich literature on reasons for vaccine rejection.^18^ However, although these arguments include those that physicians in previous studies had identified as their patients’ concerns^24^,^26^, there has yet to be an analysis of how physicians perceive the different arguments they could encounter.

There is a good reason to believe that physicians’ difficulty with rebutting arguments against vaccines would vary across different arguments. Physicians are trained to provide reassurance by giving their patients scientific facts about vaccines.^27^ This can be effective if the patient’s concern stems from a lack of knowledge, and the patient trusts the physician to provide that knowledge.^28^ However, there are many documented arguments against vaccination that scientific facts cannot directly address.^18^ Rather, these may be philosophical (eg, rejection of the epistemic basis of scientific knowledge^29^,^31^) or political (eg, rejection of vaccines along partisan political lines^32^,^34^), or may reflect an aversion to being told what to do (ie, reactance^35^). Moreover, even if a concern should in principle be assuaged with the correct knowledge, just providing facts is not always effective at dislodging misinformed beliefs.^13^ Providing facts can occasionally even backfire,^12^ particularly if an individual is motivated to interpret new information as supporting their strongly held belief.^36^ If a patient is motivated to reject a scientific counterargument, rather than explaining the science, physicians would need to address that underlying motivation to effectively deal with this type of concern.^17^ Physicians would likely find antivaccination arguments more difficult to address when the facts they have been trained to provide are insufficient as a counter.

### Attitude roots of vaccine resistance

A patient’s stated reason to reject a vaccine can be conceptualised as the manifestation, or expression, of their underlying motivations for that rejection. These are likely linked to a number of psychological factors that consistently predict vaccine hesitancy, such as conspiracist ideation (ie, the tendency to believe in conspiracy theories^3537^,^40^), distrust (eg, of healthcare systems^40^,^42^) and reactance (ie, the tendency to push back against a perceived imposition^35 40^), among others. The strength of the relationship between a certain attitude root and vaccine hesitancy may vary across countries—for example, conspiracist ideation was shown to be a consistently strong predictor of hesitancy across 24 different countries, whereas reactance was a predictor in some countries but not others.^35^ Nonetheless, these psychological factors shape and constrain people’s beliefs, attitudes and the expression of those, without the person necessarily being aware of it—leading to the terminology ‘attitude roots’ to describe these underlying motivations for people’s resistance to vaccination.^17^

In a systematic literature review, Fasce *et al*^18^ analysed over 2000 documented arguments against vaccination and identified 11 attitude roots: conspiratorial ideation, distrust, unwarranted beliefs, worldview and politics, religious concerns, moral concerns, fear and phobias, distorted risk perception, selfishness, epistemic relativism, and reactance (see table 1 for definitions and example arguments of each attitude root). This 11-root taxonomy forms a comprehensive hierarchy to group different arguments against vaccines and opens an avenue to assessing whether arguments arising from different attitude roots generate varying degrees of difficulty for rebuttal by physicians. Such an assessment would help to ascertain where there may be communication skills gaps that could be addressed with tailored training.

**Table 1:** Definition and prototypical arguments from 11 attitude roots presented in the physician survey

Attitude root	Prototypical argument
Conspiracist ideation	The authorities are lying and covering up important information about vaccines. ‘Big Pharma’ is colluding with the medical authorities to profit from people getting vaccinated. To get us vaccinated, medical authorities are spreading fear about diseases that do not exist or are fabricated.
Distrust	Medical authorities are overreacting, with vaccines being recommended for every minor illness now. Information from ‘Big Pharma’ about vaccines is not to be trusted. Healthcare authorities, politicians and governments are corrupt and profit from vaccinations.
Unwarranted beliefs	People are being offered too many vaccines nowadays, and this will overload their immune systems. Instead of vaccines, people should improve environmental factors like good hygiene, healthy lifestyles and protective measures against the disease. Scientists are still debating the benefits of vaccination, and the science is not settled.
Worldview and politics	Vaccines are just another way that the scientific elite are widening inequalities and subjugating ordinary people. Vaccinations are an expression of the inappropriate interference of the state in the freedoms of individual citizens. Politicians use vaccinations as strategies to boost their own political agendas at the expense of the common good.
Religious concerns	Vaccines interfere with God’s will: He will decide if people get the disease or not. People should abide by what religious leaders say against vaccines. The human body was created in God’s image, so it is a sin to defile it with unnatural injections.
Moral concerns	Vaccines were developed through unethical experimentation. It is our moral duty not to rely on vaccines. Parents who rely on vaccination for their child’s health demonstrate poor values.
Fears and phobias	I worry about experiencing side effects from vaccines. Vaccines contaminate the human body with toxins, heavy metals or viruses that could alter DNA. Vaccines overwhelm the immune system, especially when taken in many doses.
Distorted risk perception	Vaccinations are not needed if you live in a developed and safe country. Vaccines are riskier than the diseases themselves. Vaccination is unnecessary if you have a strong immune system that protects you from vaccine-preventable diseases.
Perceived self-interest	People do not need to be vaccinated as long as herd immunity exists. People should look after their own health rather than put themselves or their child at risk to protect others. People whose jobs allow them to adopt strong preventive measures against diseases should not need to get vaccinated.
Epistemic relativism	Vaccines are based on subjective ‘theories’ that scientists impose on people who have other equally valid perspectives. The vaccination movement does not respect other more comprehensive and holistic perspectives on health. People are experts on their own bodies so they may legitimately conclude based on their own reading that vaccination is not for them.
Reactance	Vaccination campaigns bully and harass people into getting a vaccine. People should be able to decide what goes into their bodies, so it should be a matter of free personal choice whether someone gets a vaccine. People are getting vaccinated out of ignorance and fear, according to what the nanny state expects of them.

Note. Due to a technical error, the third ‘fears and phobias’ argument was not presented in France, so the average for the ‘ffears and phobias’ root for France was calculated omitting this argument. We ran a robustness check by re-running our analyses while excluding this argument for other countries as well. Excluding this argument from the calculated averages did not substantially change the nature of the results.

### The present study

The objective of our research was to understand how difficult physicians perceived it to rebut antivaccination arguments with different attitude roots, and how the perceived difficulty of rebuttal was associated with their vaccine recommendation behaviours and their preparedness to discuss vaccines. We compared the physicians’ perceived difficulties to rebut 33 different prototypical arguments that represented each of the 11 attitude roots in a taxonomy of antivaccination argumentation.^18^ We hypothesised that physicians would report differences in the perceived difficulty of rebutting arguments from different roots. In addition, since the strength of the relationships between attitude roots and vaccine hesitancy can vary among countries,^35^ it is worth considering whether the patterns in physicians’ difficulties with arguments of different attitude roots persist across countries. The majority of studies on antivaccination arguments have been done in English, but vaccine opposition—and indeed antivaccination misinformation—is known to persist among non-English-speaking populations as well.^18^ Therefore, we could expect there to be a general correlation among different countries in how physicians perceive the difficulty of rebutting arguments, but also some country-specific differences in difficulties with certain attitude roots.

Finally, we hypothesised that greater perceived difficulty in rebutting arguments would be negatively correlated with the frequency with which physicians recommend vaccines to patients and with physicians’ ‘proactive efficacy’—defined as how prepared they felt and how proactive they were during vaccine discussions.^43^

## Materials and methods

### Participants

We collected data as part of an international cross-sectional survey on physicians’ vaccine confidence (conducted online between March and June 2022). Each country sent invitations to participate to physicians who had vaccination roles. Vaccination duties varied across countries and this is reflected in the distribution of physician types in the study (see table 2). Here, we report data (total n=2718 after excluding 157 incomplete responses to the questions investigated in this paper) from four European countries: France (n=1162), Finland (n=389), Portugal (n=560) and Germany (n=607). These countries were part of the JITSUVAX project submitted to and funded by the European Commission in 2020^44^ and represent a broad spectrum of vaccine hesitancy across member states of the European Union (EU). A current report on vaccine hesitancy among European countries revealed that 58.9%–60.4% of the general public in Germany and Finland agree that vaccines are important, safe, effective and compatible with their beliefs.^45^ This agreement was only 46.8% in France but 75.3% in Portugal. Moreover, only 67.3% of HCPs in France agreed that vaccines are important, safe, effective and compatible with their beliefs while agreement ranged between 93.9% and 95.7% in Germany, Portugal and Finland.^45^ Differences are also observable for disease burden and vaccine uptake rates. For example, Germany and France were among the five countries that accounted for 77% of all measles cases in the EU in 2022 while Finland reported a single case and Portugal no cases over the last three reporting periods.^46^ In addition, only Portugal was among the five countries that reported a coverage of ≥95% for the second dose of a measles-containing vaccine in 2021 (Germany: 93%; Finland: 93%; France: 86%). We assumed that these differences in country profiles also indicated differences in physicians’ difficulty ratings in dealing with arguments against vaccination. The demographic characteristics of the survey sample are reported in table 2. We provide analyses of the overall sample as well as per country.

**Table 2:** Demographic characteristics of the study sample

Country	France	Finland	Portugal	Germany
	(n=1162)	(n=389)	(n=560)	(n=607)
Profession				
GP	99%	67%	42%	68%
Paediatrician	<1%	29%	54%	13%
Gynaecologist	<1%	0%	0%	19%
Other role	<1%	4%	4%	0%
Gender				
Female	55%	78%	79%	38%
Male	45%	22%	21%	62%
Age (years)				
Under 40	33%	14%	68%	14%
40–49	27%	21%	19%	19%
50 and over	40%	65%	13%	68%
Vaccination status (COVID-19)				
Unvaccinated	<1%	<1%	<1%	2%
Partially vaccinated	<1%	0%	<1%	<1%
Fully vaccinated	4%	4%	7%	6%
Fully vaccinated and boosted	95%	96%	92%	92%
Vaccination status in last 3 years (Influenza)				
Not vaccinated against Influenza	4%	2%	8%	11%
At least one Influenza vaccine	96%	98%	92%	89%
Answered intention question for the following vaccines:				
COVID-19 (for adults >18 years)	0%	20%	38%	2%
COVID-19 (for pregnant women)	3%	36%	41%	10%
COVID-19 (for adolescents)	1%	21%	1%	3%
Influenza	0%	29%	45%	5%
HPV	1%	25%	1%	5%
MMR	2%	19%	1%	16%
Whooping cough	2%	53%	38%	4%

Note. We initially planned to include a UK sample, however, data quality in this sample was compromised, leaving an insufficiently large sample for comparison with the other countries (*n*n=135). Analyses with these data are available on the OSF.^76^ In each country, questions about vaccination recommendations for atarget age groups were adjusted to reflect the prevailing recommendation for those age groups in that country at the time. Details of the relevant age groups for each recommended vaccine are reported in Garrison *et al*.^47^

GPgeneral practitionerHPVhuman papillomavirusMMRmeasles, mumps & rubellaOSFOpen Science Framework

### Materials and procedure

We collected data as part of an international questionnaire on physicians’ vaccination attitudes and behaviours. Perceived difficulty to rebut antivaccination arguments was our main variable of interest. It was presented at the end of the questionnaire. Participants provided ratings of 33 arguments (3 arguments per attitude root), in response to the following question:

Below is a list of anti-vaccination arguments. All the arguments are false or misleading and have been repeatedly debunked.

Please read the messages below and indicate for each message how easy you would find it to rebut the message while interacting with a patient.

The arguments were then presented in a matrix format, in a random order for each participant. Participants responded to each argument on a 5-point Likert scale (1: I would find it very easy, 2: I would find it rather easy, 3: undecided, 4: I would find it rather difficult and 5: I would find it very difficult). The full list of prototypical arguments for each attitude root is shown in table 1. We calculated the average difficulty rating for arguments within each attitude root, as well as an overall difficulty rating across all arguments. Means and SD of ratings for all arguments by physicians in each of the countries, and correlations between each country’s average difficulty ratings of each argument as well as correlations between participants’ average difficulty rating per attitude root among the 11 attitude roots can be found in online supplemental tables S1–S5. Overall, there were strong correlations between ratings from each country (r=0.75–0.90) and for the different attitude roots (r=0.52–0.84).

The overall questionnaire included the I-Pro-VC-Be (a series of questions on vaccination recommendation behaviour, own vaccination status and determinants of vaccination attitudes^47^), and several other scale measures related to other research questions not investigated in this paper. This questionnaire was validated in all four countries^47^ and the full wording is provided asonline supplemonline supplemental file 2ental materialonline supplemental file 2. We aimed to determine the association of physicians’ difficulty rebutting arguments with physicians’ vaccine recommendation behaviours and preparedness to discuss vaccines. Therefore, we preregistered analyses with two key variables from the I-Pro-VC-Be that were targeted at measuring these validated constructs,^47^ as described below.

#### Recommendation frequency (or intentions)

Physicians responded to the question ‘*When you treat [target patient group] who have not had the [disease] vaccine, what is the percentage of these patients for whom you actively recommend the vaccine?’* seven times, once for each of the following vaccinations (and their respective target patient group in each country): MMR, HPV, whooping cough (pertussis), hepatitis-B and COVID-19 for adults, pregnant women and adolescents (aged (age range) years), respectively. The response alternatives were from 0% to 100% in 10% increments, with an option to state ‘I do not treat patients within this age/target group’ if that was the case.

For physicians who did not treat patients in the specified group, we administered a question querying their intentions to recommend the vaccine if they would treat such patients and included this response in the recommendation frequency variable instead. The intention questions were, ‘*Please imagine you are treating a (target patient) who has not had the (disease) vaccine and has no contraindications. How likely is it that you would recommend the vaccine to the patient?’* The response alternatives were from 0% to 100% in 10% increments.

We calculated the average recommendation as the mean of the recommendation frequency indicated by physicians across all vaccines.

#### Proactive efficacy

This variable measured how prepared physicians felt and how proactive they were during vaccine discussions. It comprised seven items answered on a 5-point Likert scale ((1) strongly disagree, (2) disagree, (3) undecided, (4) agree and (5) strongly agree):

- I am committed in ensuring that my patients are vaccinated.
- I am committed to keeping my knowledge about vaccination up-to-date (eg, through continuing medical education, conferences, reading).
- I am committed to developing the skills needed to communicate better with my patients about vaccination.
- I feel comfortable advising my patients about the risks and benefits of vaccines.
- I feel comfortable discussing vaccines with my patients who are highly hesitant about vaccination.
- I feel sufficiently trained and informed to discuss vaccines with all patients.
- I feel sufficiently trained on how to bring up the question of vaccines with hesitant patients.

We calculated the mean of these seven items (Cronbach’s α=0.84) as a measure of participants’ proactive efficacy.

All questionnaires were professionally translated into the relevant language of each country and checked against the English version using a back-translation protocol.

### Analysis strategy

To assess differences in difficulty to rebut arguments between countries and attitude roots, we preregistered a between-within analysis of variance on the perceived difficulty variable, with attitude root as a within-subjects factor and country as a between-subjects factor. In addition, to assess the contribution of difficulty ratings for the antivaccination arguments towards physicians’ vaccine recommendation behaviours and proactive efficacy around these behaviours, we analysed the average difficulty rating across all the arguments as a predictor of two outcome variables: average recommendation behaviour (frequency or intention) and proactive self-efficacy. For each of these outcomes, we ran a mixed-effects linear model using the lme4 package in R^48^ that included the rebuttal difficulty score as a fixed effect and country as a random effect. This model deviated slightly from our pre-registered plan to analyse the contribution of difficulty ratings for each root towards physicians’ vaccine recommendation behaviours and proactive efficacy around vaccination. The issue of multicollinearity (see online supplemental table S4) prevented use of the preregistered models. We thus used an overall rebuttal difficulty score.

### Patient and public involvement

As the relevant public group to involve in the conduct of the research, HCPs from all the participating countries were involved in designing the study questionnaire through one-on-one interviews (n=28) and a pilot test (n=272). Once the study has been published, participants will be informed of the results through a dedicated website (https://sks.to/jitsuvax).

## Results

### Arguments of different attitude roots pose different levels of difficulty for physicians

**Figure 1:**
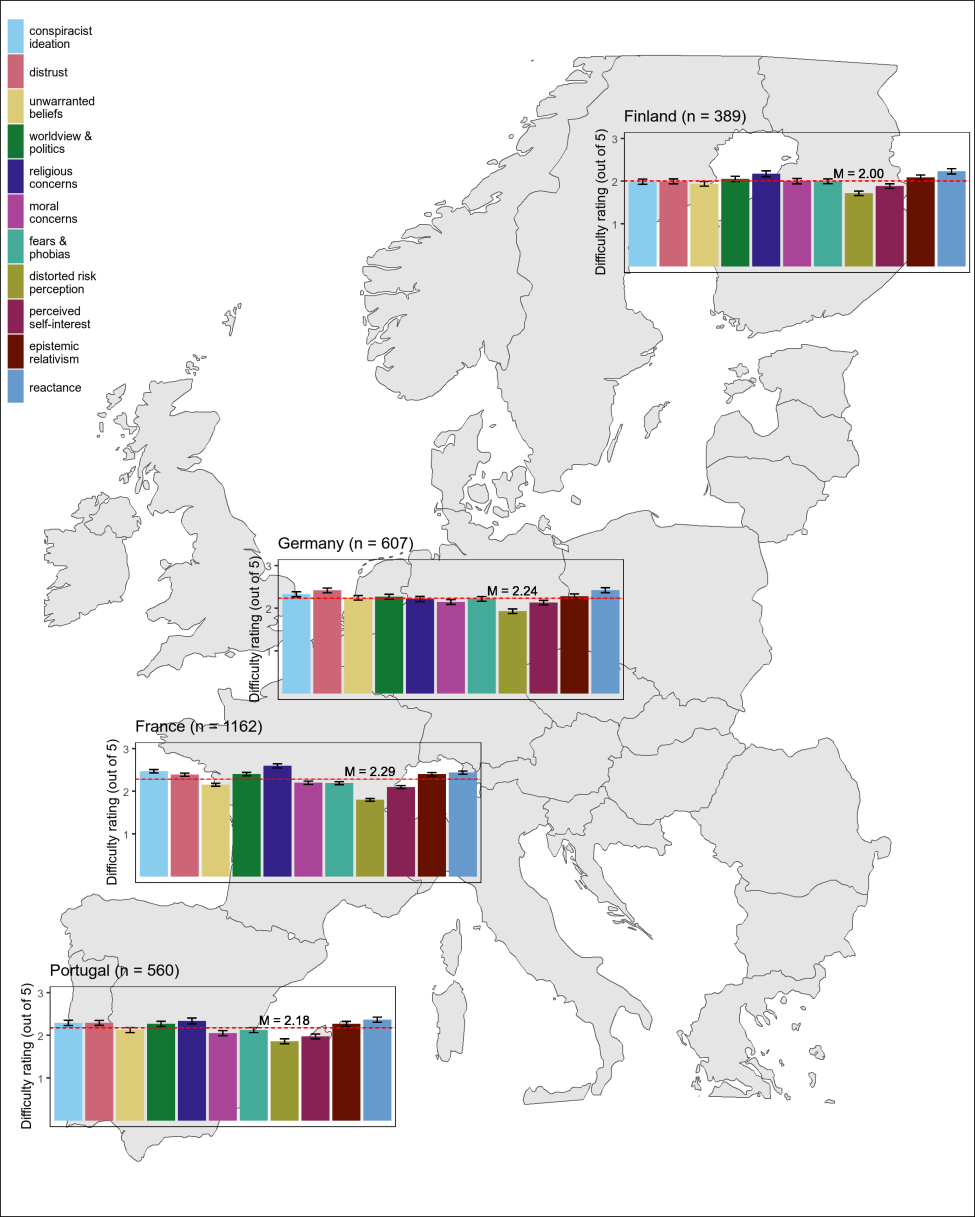
Average difficulty of rebutting arguments from each attitude root across four European Union (EU) countries.

Within each attitude root, difficulty ratings for arguments had good reliability (α>0.75). We calculated an average difficulty rating per attitude root per country. As shown in figure 1, physicians across all countries rated arguments from some roots as more difficult than others. Across the full sample, arguments based on ‘religious concerns’ and ‘reactance’ were rated as the most difficult roots to rebut, while arguments based on ‘distorted risk perception’ were the easiest. This pattern was consistent across all countries except Germany, where the most difficult attitude root to rebut was ‘distrust’.

Difficulty ratings differed significantly between attitude roots, F(7, 18137)=201.75, p<0.001, η_^p^_^2^=0.07. Difficulty ratings also varied significantly between countries F(3, 2714)=10.99, p<0.001, η_^p^_^2^=0.01. On average, French physicians indicated the most difficulty with arguments (M=2.29, SD=0.78), followed by German physicians (M=2.24, SD=0.90), Portuguese physicians (M=2.18, SD=0.98) and finally Finnish physicians (M=2.00, SD=0.78). All follow-up pairwise comparisons among countries were significant at p<0.01 after applying a Bonferroni correction (see online supplemental table S1).

The interaction between attitude root and country was significant, indicating that rebuttal difficulty across arguments varied among countries F(20, 18137)=13.41, p<0.001, η_^p^_^2^=0.02. To assess differences in difficulties with each attitude root for each country, we used one-sample t-tests to compare average ratings for each of the 11 attitude roots in a country to the mean difficulty rating of all arguments in that country. We used this approach because within each country, it is of more practical importance to know whether particular attitude roots stand out as significantly more or less difficult than the average argument difficulty in that country, rather than being concerned with specific comparisons among roots. Because this resulted in 11 comparisons per country, we applied a Bonferroni correction of α/11.

As shown in table 3, some patterns were similar across all countries: distorted risk perception arguments were on average reported as significantly easier to rebut compared with the country mean, whereas reactance arguments were on average reported as significantly more difficult to rebut compared with the country mean. French physicians showed the greatest variation in difficulty, with all mean root ratings differing significantly from the overall country mean. While the larger sample in France enabled greater power to detect significant effects, the effect sizes for the significant effects are still comparable to those of significant effects in the other countries. Physicians in Finland and Portugal also reported religious-concerns arguments as significantly more difficult and perceived self-interest arguments as significantly less difficult. German physicians reported distrust as the only other significantly more difficult (and indeed, the most difficult) attitude root.

**Table 3:** Results of follow-up one-sample t-tests comparing mean difficulty rebutting attitude roots to overall mean difficulty of rebutting arguments per country

Attitude root	France (n=1162)			Finland (n=386)			Portugal (n=560)			Germany (n=607)		
	t	P value	d	t	P value	d	t	P value	d	t	P value	d
Conspiracist ideation	**6.42**	<0.001*	**0.19**	−0.38	0.705	−0.02	2.38	0.018	0.10	2.02	0.043	0.08
Distrust	**3.92**	<0.001*	**0.11**	−0.24	0.813	−0.01	2.58	0.010	0.11	**4.34**	<0.001*	**0.18**
Unwarranted beliefs	**−5.60**	<0.001*	**−0.16**	−1.67	0.096	−0.08	−1.26	0.207	−0.05	0.18	0.856	0.01
Worldview and politics	**3.83**	<0.001*	**0.11**	1.00	0.320	0.05	1.94	0.053	0.08	0.77	0.442	0.03
Religious concerns	**8.09**	<0.001*	**0.24**	**3.02**	0.003*	**0.15**	**2.84**	0.005*	**0.12**	−0.42	0.624	−0.02
Moral concerns	**−3.30**	0.001*	**−0.10**	−0.11	0.912	−0.01	−2.75	0.006	−0.12	−2.09	0.037	−0.08
Fears and phobias	**−3.89**	<0.001*	**−0.11**	−0.20	0.844	−0.01	−1.32	0.187	−0.06	−0.45	0.651	−0.02
Distorted risk perception	**−20.57**	<0.001*	**−0.60**	**−7.25**	<0.001*	**−0.37**	**−6.80**	<0.001*	**−0.29**	**−7.10**	**<**0.001	**−0.29**
Perceived self-interest	**−7.83**	<0.001*	**−0.23**	**−2.95**	0.003*	**−0.15**	**−4.72**	<0.001*	**−0.20**	−2.58	0.010	−0.10
Epistemic relativism	**3.95**	<0.001*	**0.12**	1.75	0.081	0.09	1.92	0.056	0.08	0.95	0.340	0.04
Reactance	**5.72**	<0.001*	**0.17**	**5.24**	<0.001*	**0.27**	**4.45**	<0.001*	**0.19**	**4.31**	<0.001*	**0.17**

Note. Items in bold with p- values marked with *=significant at adjusted threshold of *p*p<0.005. Means and standard deviationsSD of all difficulty ratings per root per country are reported in (Table S2online supplemental table S2).

### Difficulty rebutting arguments is associated with lower proactive efficacy and recommendation of vaccines

Physicians who found arguments more difficult to rebut tended to recommend vaccinations significantly less, β=−0.18, p<0.001 (95% CI −0.22 to –0.15), R^2^=0.03. Physicians who perceived greater difficulty rebutting arguments also reported significantly lower proactive self-efficacy, that is, lower commitment to vaccination and self-efficacy, β=−0.23, p<0.001 (95% CI −0.26 to –0.19), R^2^=0.05. These associations remained significant in robustness checks that controlled for other I-Pro-VC-Be variables and analysed the individual recommendation and intentions variables independently (see online supplemental tables S6 and S7).

## Discussion

The physician–patient conversation is one of the most promising methods for countering vaccine hesitancy and promoting informed vaccine decisions.^1 4^ This is largely because physicians are the most trusted source of health information, and face-to-face conversations allow messages to be tailored directly to the individual.^1^ These benefits of physician–patient conversations could also be used to address a crucial barrier to informed decision-making: misinformation.^49 50^ To do this effectively, however, physicians have repeatedly called for help in dealing with misinformation.^51^ This study provides a building block for designing support measures for physician–patient conversations by identifying which types of vaccine misinformation are most difficult for physicians to deal with in different countries in Europe.

Our results indicated that physicians in Germany found it most difficult to refute arguments rooted in distrust of healthcare authorities and vaccination programmes, and arguments based on reactance compared with the country-specific mean difficulty level of arguments. The perceived difficulty in refuting arguments based on distrust and reactance could be explained by a high visibility of ‘anti-Corona’ protests in Germany. While trust in science has generally increased in Germany since the beginning of the pandemic,^52^ a significant minority including individuals with conspiracist, esoteric and extreme political views organised frequent street protests that were used to express distrust towards the government and conventional medicine.^53^ Fuelled by far-right sentiment, the distrust messages of these protests have been described as polarising and radicalising^53 54^ and may have increased the impression among physicians that it is particularly difficult to counter this form of distrust and reactance in a rational discussion.

In Finland, physicians rated religious concerns and reactance as the most difficult forms of argumentation. In contrast to the rest of the sample, distrust in health authorities and vaccination programmes was not reported to be significantly harder to rebut. This could be explained by findings from a 2021 survey on Drivers of Trust in Public Institutions, which indicated that institutional trust levels have been traditionally high in Finland.^55^ Thus, Finnish physicians may not perceive distrust in institutions as a matter of concern. However, higher difficulty ratings of arguments based on religiosity are found in Finland and also in Portugal and France. The widespread difficulty in dealing with arguments based on religious concerns in three out of four European countries may relate to religious arguments being indicative of a larger, deeper belief system that is difficult to reconcile with a physician’s scientific way of thinking.^56^ In fact, physicians often report difficulties and fears of offending patients when talking about religious issues.^57^,^59^ Moreover, research shows that debunking vaccine misinformation can be specifically challenging among highly religious individuals.^60^ Aside from religiosity, reactance is also consistently rated as more difficult than average across countries. Reactance is defined as an individual’s tendency to defend their autonomy when they experience a threat to their free behaviours.^61^ Reactance is repeatedly found to be associated with vaccine hesitancy^3562^,^64^ and during the COVID pandemic, antivaccine movements used freedom and autonomy as central arguments for their campaigns.^65 66^ These so-called health freedom movements^67^ started in the USA as opposition to vaccination mandates but have extended to antivaccine protests in Western Europe.^67^,^69^ For example, the ‘Querdenken’ (‘lateral thinking’) movement in Germany repeatedly spoke out against recommended vaccines, compulsory masks and lockdowns, basing its protest on a libertarian understanding of freedom and an emphasis on individual responsibility.^70^ Awareness about the health freedom movements and the heated debates over mandatory vaccination during the pandemic may help explain why physicians across Europe rated arguments based on reactance as a difficult challenge in consultations.

Across all four countries, arguments based on distorted risk perception, where an individual perceives that the disease is of low or inconsequential risk,^18^ were rated as the least difficult to rebut. These issues are more within the expertise of a medical doctor than, for example, religious concerns,^71^ and common sources of information for doctors such as the websites of the Robert Koch Institute in Germany or the Finnish Institute for Health and Welfare offer a variety of publications that can be consulted for such risk assessments. The reasons why differences in difficulty ratings between the countries and between arguments from different attitude roots exist remain speculative, but support the assumption of previous work that the 11 attitude roots relate to different psychological motivations.^18 41^ Understanding the patterns of difficulty with rebutting certain arguments can provide additional insights into possible problems in physician–patient conversations, for example, if there are certain psychological profiles of vaccination opposition that physicians would find more challenging.^72^ In addition, the differences and similarities in reported difficulty to rebut arguments of different attitude roots could be used to develop targeted support for physicians in their fight against misinformation.

For example, future support for physicians can target attitude roots that have been assessed by physicians in the respective country as particularly difficult to correct, with training dedicated towards understanding the motivations behind those arguments so as to better communicate with patients.^18 41 73^ A first empirical evaluation of such a tailored training revealed promising results for improving physician–patient interactions.^73^ The data from this study will allow trainers in Germany, Finland, France and Portugal to select the most relevant attitude roots for their target audience. This allows communications training to be tailored towards the actual needs of physicians in the respective country, increasing the efficiency of new training approaches. This tailoring is highly relevant given the high workload of physicians and the limited capacity for additional training.

Interestingly, results also revealed differences in overall difficulty ratings between countries. Overall, Finnish physicians rated arguments as less difficult than Portuguese, French and German physicians. Portuguese participants, in turn, rated arguments as less difficult than French and German physicians, and German physicians rated arguments as less difficult than French physicians. This pattern may reflect the scope of issues in dealing with vaccine hesitancy in different countries in the EU. Portugal is known for high vaccine uptake for a variety of vaccines,^74^ and Finland is known for traditionally high levels of population trust in institutions.^55^ Germany and France, by contrast, have been repeatedly confronted with movements and protests against vaccinations.^70 75^ These different contexts may also influence physicians’ experiences in their practices. For example, many arguments against vaccination may be hypothetical for many Portuguese physicians, whereas German and French physicians, through actual experience with vaccine hesitant patients, may perceive the difficulty of corrections to be higher. Understanding these differences between countries is important for European policy makers and health authorities to direct support where it is most needed.

The need for support is not only demonstrated by descriptive assessments of argument difficulty. Physicians who rated arguments against vaccination as more difficult to correct also showed lower self-efficacy in recommending and communicating about vaccines and lower frequencies of actually recommending vaccinations. Populations reporting higher difficulty in correcting arguments against vaccination thus also show potential for improvement in the handling of vaccination recommendations. Targeting this population and supporting them with training is a promising approach to make doctor–patient conversations more efficient. New approaches for physicians to address the 11 attitude roots have already been effectively tested and may become part of future training to improve physician–patient conversations about vaccines.^73^

### Limitations

There are some limitations to this work. First, all data are self-reported quantitative measurements. Thus, the absolute values of physicians’ difficulty ratings may be biased due to the overestimation or underestimation of their own abilities in dealing with patients’ arguments, or physicians’ discomfort in reporting difficulties refuting certain arguments about vaccination. Therefore, we mainly discuss the differences in difficulty ratings across countries and attitude roots, rather than the absolute values of the difficulty ratings. Future work could include qualitative assessments, which may bring up further insights and additional strategies physicians usually used to counter antivaccination arguments from patients.

Due to time constraints, we did not assess the frequency with which physicians encountered each argument (or one similar to it), so we cannot be sure that the difficulty ratings were not an indirect proxy of the frequency with which physicians are confronted with certain arguments. Physicians also did not indicate the type of rapport they have with their patients, which may also affect how difficult they find it to counter certain arguments. The results of this study can, therefore, only be a building block to assess which attitude roots are most relevant for the day-to-day work of physicians in the four European countries, and future work may wish to assess other factors that could potentially affect physicians’ difficulty and boost their self-efficacy in rebutting antivaccination arguments.

Finally, our sample of physicians was recruited through convenience sampling in only the four targeted countries, and over-represented general practitioners, which may have meant we do not represent all doctors in each country. It would be good for future research to extend our findings with wider groups of HCPs (eg, nurses, midwives) who also have vaccination roles, and in other countries. Studying HCPs who may also specialise in different subgroups of patients could also give an indication of what arguments HCPs find harder to rebut among these patient groups.

## Conclusions

Physicians are the public’s most trusted source of health information. Thus, understanding the difficulties of physicians in dealing with misinformation is a key step to promote informed decision-making among patients in Europe. In this study, we found that physicians in four European countries showed varying degrees of difficulty debunking arguments against vaccination, depending on which of the 11 psychological roots of misinformation the arguments are based on. The resulting country-specific profiles of difficulty ratings by physicians can help to better tailor future educational materials to the needs of physicians in Europe. A website with tailored answers for physicians for each of the 11 attitude roots can be found at https://jitsuvax.info, providing accessible online resources for self-directed learning by physicians who may not have opportunities to access formalised vaccine training programmes.

## supplementary material

10.1136/bmjph-2023-000195 online supplemental file 1

10.1136/bmjph-2023-000195 online supplemental file 2

## Data Availability

Data are available in a public, open access repository.
