# Supplementary material for: Difficulties faced by physicians from four European countries in rebutting antivaccination arguments: a cross-sectional study
Source: BMJ Public Health. 2024 Mar 12;2(1):e000195. doi: 10.1136/bmjph-2023-000195 (PMC11812751; doi:10.1136/bmjph-2023-000195)
Supplement: online supplemental file 1 [file bmjph-2-1-s001.pdf]

Supplementary Information

This document provides Supplementary Information to the manuscript "Difficulties faced by physicians from four European countries in rebutting anti-vaccination arguments".

Mean argument difficulty ratings across four countries

Table S1 shows the mean difference in difficulty ratings between countries and the associated *p*-value in pairwise between-country comparisons. Table S2 shows the means (and standard deviations) of all difficulty ratings for each of the 33 arguments by HCPs in the four countries (France, Finland, Portugal, and Germany) and Table S3 shows the means (and standard deviations) of average difficulty ratings per attitude root, calculated across the three arguments per root. Arguments are ordered in the same way as Table 1 in the main manuscript, with the attitude root for each argument given here to facilitate cross-checking of the text of the argument.

Table S1

Between-country pairwise comparisons of HCPs' perceived difficulty in rebutting arguments

Country pair	Mean difference	*p-value**
France-Finland	0.28	$< .001$
France-Portugal	0.11	$< .001$
France-Germany	0.05	.009
Finland-Portugal	-0.17	$< .001$
Finland-Germany	-0.23	$< .001$
Portugal-Germany	-0.06	.005

**p*-values are Bonferroni-adjusted for multiple comparisons.

Table S2

Means and standard deviations of argument difficulty ratings across four countries

Attitude root	Argument	France	Finland	Portugal	Germany
	1	2.58 (1.17)	1.99 (1.05)	2.36 (1.27)	2.41 (1.26)
Conspiracist ideation	2	2.63 (1.16)	2.09 (1.05)	2.43 (1.26)	2.40 (1.22)
	3	2.20 (1.13)	1.87 (1.02)	2.08 (1.25)	2.18 (1.24)
	1	2.07 (0.98)	1.92 (0.92)	2.14 (1.19)	2.23 (1.12)
Distrust	2	2.54 (1.08)	2.08 (0.96)	2.32 (1.16)	2.51 (1.17)
	3	2.55 (1.17)	1.97 (1.05)	2.42 (1.26)	2.51 (1.27)
	1	2.03 (1.00)	1.92 (0.94)	1.97 (1.19)	2.05 (1.12)
Unwarranted beliefs	2	2.36 (1.02)	1.97 (0.85)	2.33 (1.18)	2.35 (1.13)
	3	2.06 (1.05)	1.92 (0.93)	2.07 (1.16)	2.33 (1.16)
	1	2.31 (1.20)	2.02 (1.07)	2.17 (1.22)	2.12 (1.21)
Worldview & politics	2	2.43 (1.17)	2.05 (1.04)	2.33 (1.30)	2.30 (1.26)
	3	2.46 (1.17)	2.08 (1.05)	2.30 (1.22)	2.38 (1.23)
	1	2.64 (1.42)	2.20 (1.20)	2.39 (1.44)	2.23 (1.37)
Religious concerns	2	2.53 (1.37)	2.15 (1.17)	2.30 (1.41)	2.25 (1.28)
	3	2.62 (1.43)	2.16 (1.20)	2.32 (1.41)	2.16 (1.34)
	1	2.14 (1.08)	2.13 (1.03)	2.12 (1.18)	2.18 (1.23)
Moral concerns	2	2.40 (1.25)	1.99 (1.11)	2.11 (1.30)	2.18 (1.78)
	3	2.05 (1.04)	1.87 (1.03)	1.92 (1.21)	2.07 (1.21)
	1	2.56 (0.96)	2.16 (0.90)	2.38 (1.12)	2.53 (1.17)
Fear & phobias	2	2.12 (1.09)	1.92 (1.01)	2.04 (1.23)	2.13 (1.23)
	3	-	1.90 (0.92)	1.94 (1.17)	1.99 (1.10)
	1	1.80 (0.93)	1.64 (0.85)	1.88 (1.18)	1.90 (1.16)
Distorted risk perception	2	1.68 (0.90)	1.64 (0.86)	1.78 (1.20)	1.86 (1.12)
	3	1.91 (0.97)	1.86 (0.94)	1.93 (1.18)	2.03 (1.16)
	1	1.97 (0.95)	1.76 (0.88)	1.91 (1.17)	2.10 (1.11)
Perceived self-interest	2	2.22 (1.04)	1.96 (0.96)	2.05 (1.14)	2.18 (1.18)
	3	2.10 (1.00)	1.92 (0.93)	1.96 (1.17)	2.11 (1.15)
	1	2.27 (1.15)	1.99 (1.01)	2.13 (1.24)	2.18 (1.22)
Epistemic relativism	2	2.54 (1.13)	2.17 (0.99)	2.38 (1.21)	2.39 (1.17)
	3	2.39 (1.16)	2.08 (1.04)	2.29 (1.28)	2.26 (1.23)
	1	2.35 (1.04)	2.04 (0.94)	2.18 (1.18)	2.27 (1.22)
Reactance	2	2.66 (1.14)	2.63 (1.13)	2.77 (1.27)	2.74 (1.26)
	3	2.30 (1.11)	2.02 (1.06)	2.14 (1.20)	2.25 (1.23)

Note. France did not complete ratings for the third argument in the "Fears & phobias" root.

Table S3

Means and standard deviations of argument difficulty per attitude root and on average for all arguments across four countries

Attitude root	France	Finland	Portugal	Germany
Conspiracist ideation	2.47 (1.17)	1.99 (1.04)	2.29 (1.27)	2.33 (1.24)
Distrust	2.39 (1.10)	1.99 (0.98)	2.29 (1.21)	2.42 (1.19)
Unwarranted beliefs	2.15 (1.03)	1.94 (0.91)	2.12 (1.19)	2.24 (1.14)
Worldview & politics	2.40 (1.18)	2.05 (1.05)	2.27 (1.25)	2.27 (1.23)
Religious concerns	2.60 (1.41)	2.17 (1.19)	2.33 (1.42)	2.21 (1.33)
Moral concerns	2.20 (1.14)	2.00 (1.06)	2.05 (1.23)	2.14 (1.21)
Fear & phobias	2.19 (1.03)	2.00 (0.95)	2.12 (1.19)	2.22 (1.19)
Distorted risk perception	1.80 (0.94)	1.71 (0.89)	1.86 (1.19)	1.93 (1.15)
Perceived self-interest	2.10 (1.01)	1.88 (0.93)	1.97 (1.16)	2.23 (1.15)
Epistemic relativism	2.40 (1.15)	2.08 (1.01)	2.27 (1.25)	2.28 (1.21)
Reactance	2.44 (1.11)	2.23 (1.08)	2.36 (1.25)	2.42 (1.25)
Overall	2.29 (0.78)	2.00 (0.78)	2.18 (0.98)	2.24 (0.90)

Note. Argument difficulty per attitude root was a composite average of the three argument ratings per root.

Correlations among attitude roots and countries

Table S4 gives all of the bivariate correlations between difficulty ratings for each pair of attitude roots, calculated based on individual responses from the entire sample (n = 2,718). As shown in the table, the difficulty ratings of the attitude roots were highly correlated. All correlations were significant at $p < .001$.

We also calculated correlations between countries for difficulty ratings of the 33 arguments. These correlations, shown in Table S5, were based on each country's average difficulty rating (across participants in that country) for each of the 33 arguments. All the bivariate correlations between countries for these 33 average ratings were significantly correlated (at $p < .001$).

Table S4

Correlations among argument difficulty ratings for the 11 attitude roots

	Conspiracist ideation	Distrust	Unwarranted beliefs	Fears & phobias	Distorted risk perception	Worldview & politics	Religious concerns	Moral concerns	Perceived self-interest	Epistemic relativism
Distrust	0.84	-								
Unwarranted beliefs	0.72	0.76	-							
Fears & phobias	0.66	0.70	0.74	-						
Distorted risk perception	0.67	0.69	0.75	0.76	-					
Worldview & politics	0.81	0.79	0.71	0.68	0.73	-				
Religious concerns	0.65	0.60	0.52	0.52	0.60	0.73	-			
Moral concerns	0.73	0.72	0.71	0.70	0.78	0.81	0.72	-		
Perceived self-interest	0.70	0.72	0.75	0.75	0.84	0.76	0.63	0.79	-	
Epistemic relativism	0.74	0.72	0.71	0.69	0.77	0.81	0.70	0.80	0.81	-
Reactance	0.73	0.74	0.69	0.69	0.69	0.79	0.63	0.73	0.75	0.77

Note. All correlations in the table were significant at $p < .001$. Each bivariate correlation was calculated between two different attitude root scores, which were each a composite average of three argument ratings per root.

Table S5

Correlations among argument difficulty ratings for the four countries

	France	Finland	Portugal
Finland	0.79	-	
Portugal	0.90	0.88	-
Germany	0.75	0.77	0.90

Note. All correlations in the table were significant at $p < .001$. Each bivariate correlation was calculated between

Robustness checks

We conducted robustness checks on the associations between argument difficulty and recommendation frequency and intentions variables separately. Higher perceived argument difficulty was significantly associated with lower recommendation frequency, β = -0.18, $p < .001$. Higher perceived argument difficulty was also significantly associated with lower recommendation intentions, β = -0.11, $p = .001$.

We also conducted robustness checks on the linear mixed effects regressions with argument difficulty as a predictor and country as a random effect, and controlling for nine constructs from the I-Pro-VC-Be questionnaire (Garrison et al., 2023): perceived vaccine safety, complacency (about diseases), risk-benefit perception for vaccines, collective responsibility, trust in health authorities, constraints to recommending vaccines, reluctant trust in authorities, perceived professional norms, and openness to patients delaying vaccination. Table S6 shows the regression coefficients for each of these models, predicting recommendation behaviour (as a combined recommendation and intention variable) and proactive self-efficacy. Table S7 shows the regression coefficients when the models are run on recommendation frequency and intentions as separate measures.

Table S6

Standardised coefficients in linear mixed-effects regressions with argument difficulty predicting recommendation behaviour and proactive efficacy

	Recommendation behaviour			Proactive efficacy		
Effect	Standardised coefficient	*p*-value	95% CI	Standardised coefficient	*p*-value	95% CI
Argument difficulty	-0.07	$< .001$	-0.10, -0.03	-0.14	$< .001$	-0.18, -0.11
Vaccine safety	0.04	.028	0.01, 0.08	0.01	.794	-0.03, 0.04
Complacency	-0.13	$< .001$	-0.17, -0.09	-0.02	.423	-0.06, 0.02
Risk-benefit	0.26	$< .001$	0.22, 0.31	0.20	$< .001$	0.16, 0.24
Collective	0.13	$< .001$	0.10, 0.17	0.15	$< .001$	0.11, 0.19
Trust	0.10	$< .001$	0.06, 0.14	0.07	$< .001$	0.03, 0.12
Constraints	0.03	.083	-0.003, 0.06	-0.001	.932	-0.04, 0.03
Reluctant trust	-0.06	$< .001$	-0.09,-0.03	-0.17	$< .001$	-0.20, -0.13
Professional norms	-0.03	.073	-0.07, 0.003	0.08	$< .001$	0.05, 0.12
Openness to patients	-0.03	.135	-0.06, 0.01	0.16	$< .001$	0.12, 0.19

Table S7

Standardised coefficients in linear mixed-effects regressions with argument difficulty predicting recommendation frequency and intentions as independent measures

	Recommendation frequency (n = 2711)			Recommendation intentions (n = 820)		
Effect	Standardised coefficient	p-value	95% CI	Standardised coefficient	p-value	95% CI
Argument difficulty	-0.06	$<$ .001	-0.10, -0.03	-0.05	.103	-0.12, 0.01
Vaccine safety	0.05	.019	0.01, 0.08	0.06	.145	-0.02, 0.15
Complacency	-0.13	$<$ .001	-0.16, -0.09	-0.12	.005	-0.21, -0.04
Risk-benefit	0.26	$<$ .001	0.21, 0.30	0.15	.100	0.06, 0.24
Collective	0.14	$<$ .001	0.10, 0.17	-0.06	.096	-0.14, 0.01
Trust	0.09	$<$ .001	0.05, 0.14	0.06	.193	-0.03, 0.16
Constraints	0.03	.102	-0.01, 0.06	0.05	.145	-0.02, 0.12
Reluctant trust	-0.06	.001	-0.09, -0.02	-0.10	.003	-0.17, -0.04
Professional norms	-0.03	.095	-0.06, 0.01	-0.06	.112	-0.12, 0.02
Openness to patients	-0.01	.438	-0.05, 0.02	-0.05	.167	-0.12, 0.02

Subgroup differences in difficulty ratings

We present here descriptive differences in difficulty ratings between demographic subgroups among the physicians in our data. Table S8 shows the comparisons between each subgroup per country. Figure S1 shows there are differences between subgroups overall, but the patterns in difficulty across the attitude roots remain similar. Exploratory pairwise comparisons between each of the subgroups indicated that female physicians reported more difficulty than male physicians in rebutting arguments, $p < .001$; GPs reported more difficulty than non-GPs, $p < .001$; and physicians aged under 40 years reported more difficulty than physicians aged over 50 years, though this difference was not significant once a Bonferroni was applied, $p = .060$. There was no significant difference in reported difficulty between physicians with different flu vaccination status, $p = .082$. Too few physicians in the overall sample were not fully vaccinated for COVID-19 (see Table 1 in main paper) to allow a sensible comparison between these groups.

Regarding these differences, research suggests that patients trust older physicians more which could make it easier for them to discuss vaccines with patients (Murray & McCrone, 2015). Moreover, research suggests that older people are more confident about the accuracy of their own beliefs (Prims & Moore, 2017). This could cause increased confidence in dealing with any kind of argument. However, reasons for this pattern remain speculative. Certain groups of physicians may be less keen to report difficulties or equally, some groups could be overconfident in their own abilities.

Table S8

Mean (and standard deviation) of difficulty rebutting arguments by gender, age group, profession, and vaccination status of physicians in four countries

Subgroup	France (n = 1162)		Finland (n = 389)		Portugal (n = 560)		Germany (n = 607)	
	n	Mean difficulty (SD)	*n*	Mean difficulty (SD)	*n*	Mean difficulty (SD)	*n*	Mean difficulty (SD)
Male	520	2.19 (0.82)	86	1.94 (0.82)	116	2.15 (1.13)	375	2.13 (0.88)
Female	638	2.36 (0.74)	303	2.02 (0.77)	444	2.18 (0.94)	228	2.39 (0.91)
Aged < 40 yrs	384	2.35 (0.75)	55	2.23 (0.58)	378	2.15 (0.91)	86	2.40 (0.90)
Aged 40-49 yrs	311	2.23 (0.72)	81	2.02 (0.63)	108	2.19 (1.05)	116	2.24 (0.97)
Aged > 50 yrs	467	2.27 (0.84)	253	1.95 (0.85)	74	2.28 (1.23)	405	2.20 (0.88)
GP	1156	2.29 (0.78)	262	2.02 (0.76)	236	2.21 (0.94)	412	2.31 (0.93)
Non-GP*	-	-	127	1.96 (0.82)	324	2.15 (1.02)	195	2.07 (0.82)
Not fully vaccinated against COVID-19*	-	-	-	-	-	-	15	3.34 (0.59)
Fully vaccinated against COVID-19**	1154	2.28 (0.78)	388	2.00 (0.78)	554	2.17 (0.98)	592	2.21 (0.89)
Number of Flu vaccines in last 3 years								
None*	46	2.42 (0.80)	-	-	45	2.39 (1.13)	67	2.27 (0.79)
At least 1	1116	2.28 (0.78)	381	2.01 (0.78)	515	2.16 (0.97)	540	2.23 (0.91)

Note. *Categories with fewer than 10 responses are excluded. **Fully vaccinated includes those who received a full course of the vaccine as well as those who received additional booster doses.

Figure S1

Average difficulty of rebutting arguments from each attitude root by gender, age group, and profession of physicians.

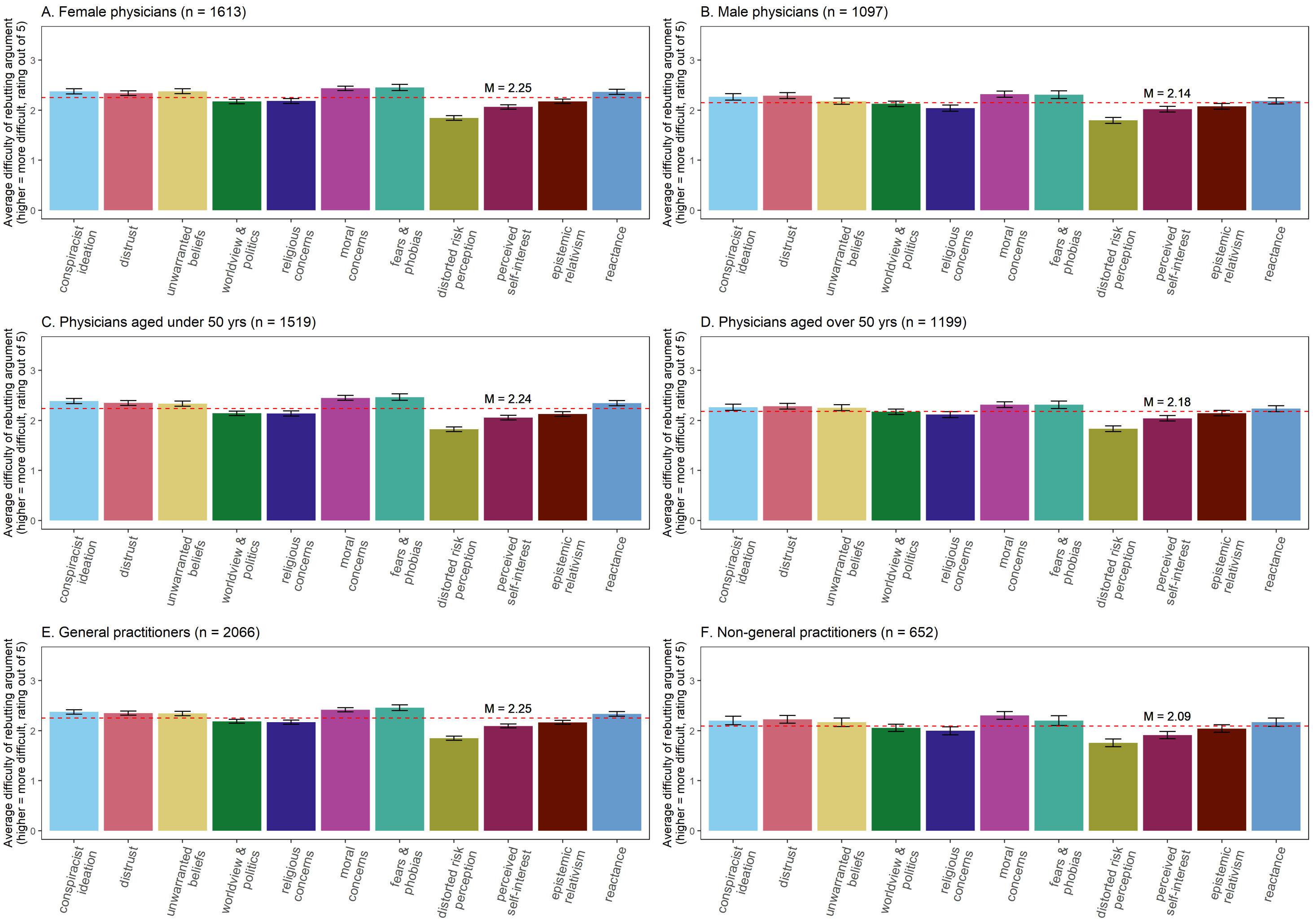

Figure S2

Average difficulty of rebutting arguments from each attitude root by vaccination status of physicians.

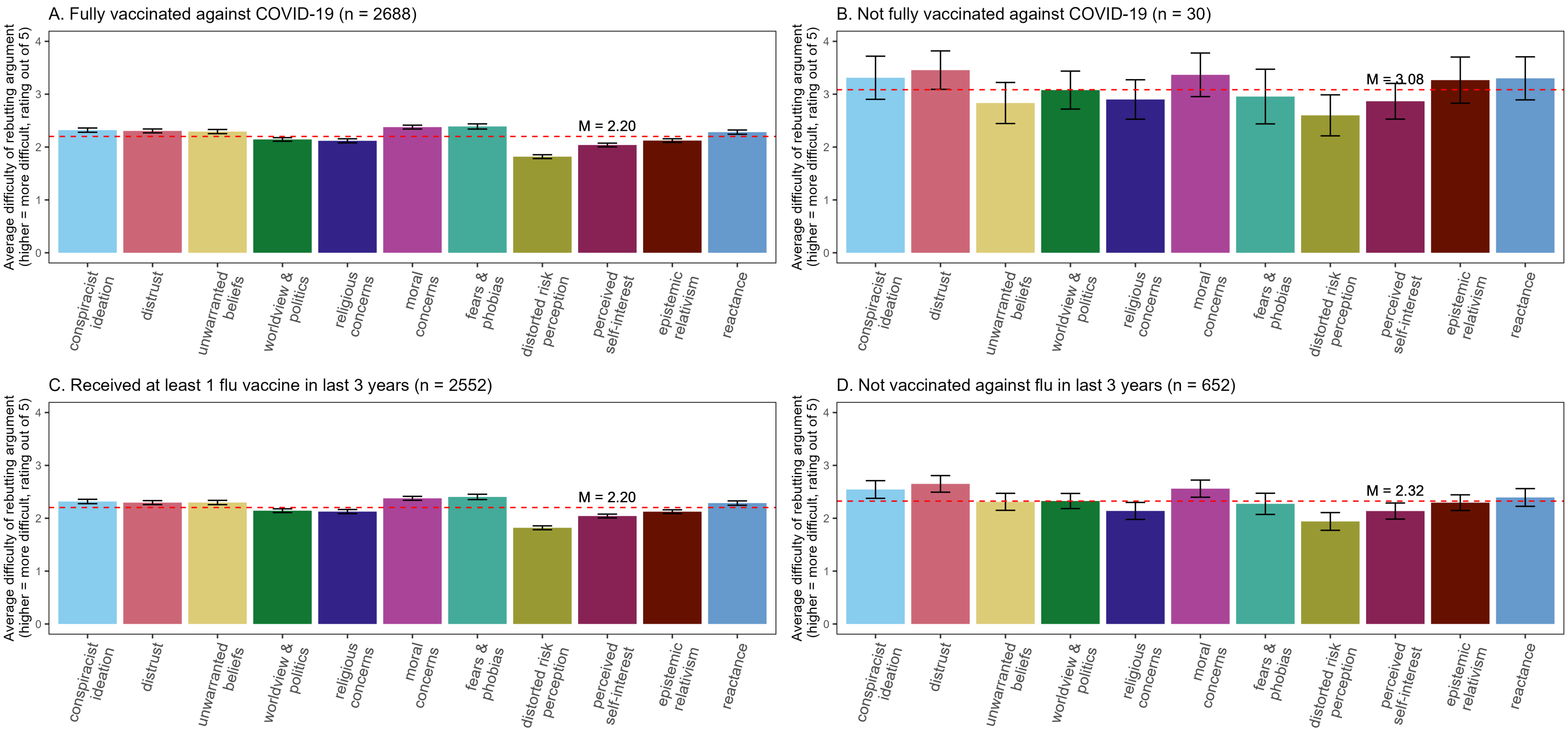

References

Garrison, A., Karlsson, L., Fressard, L., Fasce, A., Rodrigues, F., Schmid, P., . . . Verger, P. (2023). nternational adaptation and validation of the Pro-VC-Be: measuring the psychosocial determinants of vaccine confidence in healthcare professionals in European countries. *Expert Review of Vaccines*, *22*(1), 726-737.

Murray, B., & McCrone, S. (2015). An integrative review of promoting trust in the patient-primary care provider relationship. *Journal of Advanced Nursing*, *71*(1), 3–23. doi: 10.1111/jan.12502

Prims, J. P., & Moore, D. A. (2017). Overconfidence over the lifespan. *Judgment and decision making*, *12*(1), 29–41.
