# Supplementary material for: Difficulties faced by physicians from four European countries in rebutting antivaccination arguments: a cross-sectional study
Source: BMJ Public Health. 2024 Mar 12;2(1):e000195. doi: 10.1136/bmjph-2023-000195 (PMC11812751; doi:10.1136/bmjph-2023-000195)
Supplement: online supplemental file 2 [file bmjph-2-1-s002.pdf]

Study Questionnaire
I-Pro-VC-Be

Text within brackets (“[xxx]”) were adapted to the context (e.g., official vaccine recommendations) in each country that the questionnaire was administered.

All questions were back-translated into the relevant language(s) in each country and pre-tested using cognitive interviews and pilot testing with HCPs prior to roll-out for the study.

I-Pro-VC-Be variables analysed in the study are indicated in blue and marked with ^.

VACCINATION BEHAVIOR

General recommendation behaviour

For the patients you treat and for whom vaccinations are relevant according to official guidelines:
1. How often do you check that your patients have received the vaccines recommended for them (whether from records or asking)?
2. How often do you bring up the subject of vaccination?
3. How often do you recommend the vaccines that are indicated for them?

Response alternatives for question 1–3:

a) Never
b) Sometimes
c) Often
d) Always

^Recommendation frequency and recommendation intentions

The subquestion (lowercase letter) was only shown if participant responds “I do not treat patients within this age/target group” to the first question (uppercase letter).

For each situation, suppose you have all of the documentation/information necessary to know the vaccination status of the patient mentioned.

A. When you treat mothers [who have just given birth] and who have not had the whooping cough vaccine, what is the percentage of these patients for whom you actively recommend the vaccine?
(a. Please imagine you are treating a mother [who has just given birth], who has not had the whooping cough vaccine, and has no contraindications. How likely is it that you would recommend the vaccine to the patient?)

B. When you treat young [girls and boys aged <insert age range>] who have not had the human papilloma virus vaccine, what is the percentage of these patients for whom you actively recommend the vaccine?

(b. Please imagine you are treating a young [girl and boy aged <insert age range>] who has not had the human papilloma virus vaccine and has no contraindications. How likely is it that you would recommend the vaccine to the patient?)

C. When you treat adults over [<insert age>] years old who have not had the seasonal flu vaccine, what is the percentage of these patients for whom you actively recommend the vaccine?
(c. Please imagine you are treating an adult over [<insert age>] years old who has not had the seasonal flu vaccine and has no contraindications. How likely is it that you would recommend the vaccine to the patient?)

D. When you treat adults who have not had the Covid-19 vaccine, what is the percentage of these patients for whom you actively recommend the vaccine?
(d. Please imagine you are treating an adult who has not had the Covid-19 vaccine and has no contraindications. How likely is it that you would recommend the vaccine to the patient?)

E. When you treat [<insert age range>] old adolescents who have not had the Covid-19 vaccine, what is the percentage of these patients for whom you actively recommend the vaccine?
(e. Please imagine you are treating a [<insert age range>] old adolescent who has not had the Covid-19 vaccine and has no contraindications. How likely is it that you would recommend the vaccine to the patient?)

F. When you treat pregnant women who have not had the Covid-19 vaccine, what is the percentage of these patients for whom you actively recommend the vaccine?
(f. Please imagine you are treating a pregnant woman who has not had the Covid-19 vaccine and has no contraindications. How likely is it that you would recommend the vaccine to the patient?)

G. When you treat infants who have not had the MMR vaccine, what is the percentage of these patients for whom you actively recommend the vaccine?
(g. Please imagine you are treating an infant who has not had the MMR vaccine and has no contraindications. How likely is it that you would recommend the vaccine to the patient?)

H. When you treat [<insert age range>] old children who have not had the Covid-19 vaccine, what is the percentage of these patients for whom you actively recommend the vaccine?
(h. Please imagine you are treating a [<insert age limit>] old children who has not had the Covid-19 vaccine and has no contraindications. How likely is it that you would recommend the vaccine to the patient?)

Response scale for frequency questions (uppercase letters):
I do not treat patients within this age/target group

a) 0% - I do not actively recommend it to any of these patients
b) 10%
c) 20%
d) 30%
e) 40%
f) 50%

g) 60%
h) 70%
i) 80%
j) 90%
k) 100% - I actively recommend it to all of these patients

Response scale for recommendation intentions (lowercase letters):

a) 0% - I would never recommend it
b) 10%
c) 20%
d) 30%
e) 40%
f) 50%
g) 60%
h) 70%
i) 80%
j) 90%
k) 100% - I would certainly recommend it

Personal vaccinations

1. How many times have you been vaccinated against influenza during the last three years?

a) 0
b) 1
c) 2
d) 3

2. Have you been vaccinated against Covid-19?

a) No
b) Yes, I am partially vaccinated ([one dose of the Pfizer-BioNTech, Moderna, or Oxford/AstraZeneca vaccine])
c) Yes, I am fully vaccinated ([two doses of the Pfizer-BioNTech, Moderna, or Oxford/AstraZeneca vaccine, or one dose of the Johnson & Johnson vaccine])
d) Yes, I am fully vaccinated and received a booster ([third dose of Pfizer-BioNTech or Moderna, or a second dose of the Johnson & Johnson vaccine])

DETERMINANTS OF VACCINATION BEHAVIOR

The following response scale applies to all questions in the Determinants of vaccination behaviour section:

a) Strongly disagree
b) Somewhat disagree
c) Undecided
d) Somewhat agree
e) Strongly agree

Vaccine risk perception
1. Vaccines against measles are safe
2. Vaccines against influenza are safe
3. Vaccines against hepatitis B are safe
4. Vaccines against human papillomaviruses are safe
5. Vaccines against Covid-19 in my country are safe

Complacency
6. Today, some vaccines recommended by [<insert relevant authority>] are not useful, because the diseases they prevent are not serious
7. Children are vaccinated against too many diseases
8. Children are vaccinated at too young an age

Benefit/risk balance perception
The following statements refer to the benefit/risk balance within the targeted population for each vaccine (e.g. the measles vaccine for infants).

9. The benefits of the vaccine against measles outweigh its potential risks
10. The benefits of the vaccine against influenza outweigh its potential risks
11. The benefits of the vaccine against hepatitis B outweigh its potential risks
12. The benefits of the vaccine against human papillomaviruses outweigh its potential risks
13. The benefits of the vaccines against Covid-19 available in my country outweigh their potential risks

Perceived importance of collective responsibility
14. I recommend the vaccines on the vaccination schedule to my patients because it's essential to contribute to the protection of the population (community immunity)
15. I recommend the vaccines in the official schedule to my hesitant patients, explaining to them the importance of community immunity

Confidence in authorities
16. I trust the information provided by the [<insert relevant authority>] about the risks and benefits of vaccines
17. I trust the [<insert relevant authority>] to establish the vaccination strategy
18. I trust the [<insert relevant authority>] to ensure that vaccines are safe

^Commitment to vaccination
19. I am committed in ensuring that my patients are vaccinated.
20. I am committed to keeping my knowledge about vaccination up-to-date (e.g. through CME, conferences, reading)
21. I am committed to developing the skills needed to communicate better with my patients about vaccination

^Self-efficacy
22. I feel comfortable advising my patients about the risks and benefits of vaccines
23. I feel comfortable discussing vaccines with my patients who are highly hesitant about vaccination
24. I feel sufficiently trained and informed to discuss vaccines with all patients

25. I feel sufficiently trained on how to bring up the question of vaccines with hesitant patients

Openness to patients
26. Patients who are hesitant about the benefits and risks of vaccines have legitimate questions
27. I inform my patients about the benefits and risks of vaccines without trying to influence them
28. I am open to patients delaying immunization of their children

Perceived constraints
29. The cost of some vaccines is a problem for some patients and can keep me from prescribing them
30. The lack of availability of certain vaccines in my country is sometimes a problem that can keep me from prescribing them to my patients
31. The lack of availability of certain vaccines in my place of work is sometimes a problem that can keep me from prescribing them to my patients

Reluctant trust
32. I may sometimes recommend vaccines from the official schedule even if I feel I am not sufficiently informed
33. I may sometimes recommend vaccines from the official schedule even if I feel the vaccination policy is not sufficiently clear
34. I may sometimes recommend the vaccines on the official schedule even in cases where I have doubts about their safety

Difficulty of rebutting anti-vaccination arguments
^These questions were all included in the analysis.

Below is a list of anti-vaccination arguments. All the arguments are false or misleading and have been repeatedly debunked.

Please read the messages below and indicate for each message how easy you would find it to rebut the message while interacting with a patient.

Response alternatives:

a) I would find it very easy
b) I would find it rather easy
c) Undecided
d) I would find it rather difficult
e) I would find it very difficult

1. The authorities are lying and covering up important information about vaccines.
2. “Big Pharma” is colluding with the medical authorities to profit from people getting vaccinated.
3. To get us vaccinated, medical authorities are spreading fear about diseases that do not exist or are fabricated.

4. Medical authorities are overreacting, with vaccines being recommended for every minor illness now.
5. Information from “Big Pharma” about vaccines is not to be trusted.
6. Healthcare authorities, politicians, and governments are corrupt and profit from vaccinations.
7. People are being offered too many vaccines nowadays, and this will overload their immune systems.
8. Instead of vaccines, people should improve environmental factors like good hygiene, healthy lifestyles, and protective measures against the disease.
9. Scientists are still debating the benefits of vaccination, and the science is not settled.
10. Vaccines are just another way that the scientific elite are widening inequalities and subjugating ordinary people.
11. Vaccinations are an expression of the inappropriate interference of the state in the freedoms of individual citizens.
12. Politicians use vaccinations as strategies to boost their own political agendas at the expense of the common good.
13. Vaccines interfere with God’s will: He will decide if people get the disease or not.
14. People should abide by what religious leaders say against vaccines.
15. The human body was created in God’s image, so it is a sin to defile it with unnatural injections.
16. Vaccines were developed through unethical experimentation.
17. It’s our moral duty not to rely on vaccines
18. Parents who rely on vaccination for their child's health demonstrate poor values.
19. I worry about experiencing side effects from vaccines.
20. Vaccines contaminate the human body with toxins, heavy metals or viruses that could alter DNA.
21. Vaccines overwhelm the immune system, especially when taken in many doses.
22. Vaccinations are not needed if you live in a developed and safe country.
23. Vaccines are riskier than the diseases themselves.
24. Vaccination is unnecessary if you have a strong immune system that protects you from vaccine-preventable diseases.
25. People do not need to be vaccinated as long as herd immunity exists.
26. People should look after their own health rather than put themselves or their child at risk to protect others.
27. People whose jobs allow them to adopt strong preventive measures against diseases should not need to get vaccinated.
28. Vaccines are based on subjective “theories” that scientists impose on people who have other equally valid perspectives.
29. The vaccination movement does not respect other more comprehensive and holistic perspectives on health.
30. People are experts on their own bodies so they may legitimately conclude based on their own reading that vaccination is not for them.
31. Vaccination campaigns bully and harass people into getting a vaccine.
32. People should be able to decide what goes into their bodies, so it should be a matter of free personal choice whether someone gets a vaccine.
33. People are getting vaccinated out of ignorance and fear, according to what the nanny state expects of them.
